# Are Oligodendrocytes the Culprits or Victims in Alzheimer’s Disease

**DOI:** 10.33549/physiolres.935451

**Published:** 2025-04-01

**Authors:** Deepthi RAPAKA, Arthur SANIOTIS, Maciej HENNEBERG, Veera Raghavulu BITRA

**Affiliations:** 1 Department of Pharmacy, DDT College of Medicine, Gaborone, Botswana; 2 Bachelor of Doctor Assistance Department, DDT College of Medicine, Gaborone, Botswana; 3 Biological Anthropology and Comparative Anatomy Research Unit, School of Biomedicine, University of Adelaide, Adelaide, Australia; 4 Institute of Evolutionary Medicine, University of Zürich, Zürich, Switzerland; 5 School of Pharmacy, Faculty of Health Sciences, University of Botswana, Gaborone, Botswana

**Keywords:** Oligodendrocytes, Myelination, Alzheimer’s disease, Glial cells

## Abstract

Oligodendrocytes are vital for the functioning of the nervous system. Oligodendrocyte-created myelin sheaths work as dynamic partners which play a substantial role in the myelination of axons. In addition to its well-known functions of providing insulation and enhancing conduction velocity, myelination controls axons’ maturity, longevity, and regenerative ability *via* trophic support and signalling molecules. Myelination also regulates ion concentration and offers neuroprotection. Myelin is generated *via* complex procedures including cell differentiation, specialised lipids, and protein synthesis. Understanding the physiology of myelin sheath formation is required to understand various neurological disorders associated with myelin sheath damage. This review focuses on our growing understanding of the intricate actions and changes in oligodendrocytes during the course of evolution and in Alzheimer’s disease.

## History and oligodendrocyte alteration in human evolution

Although there is a plethora of research regarding the function of oligodendrocytes (OLs) in human brain development and maintenance, the evolution of OLs in the hominin clade remains unclear [1]. It has long been assumed that since OLs are involved in axonal myelination, this function would have been primarily selected by natural selection. However, this idea is speculative. It has been proposed that OLs in chordate ancestors may have performed various other functions apart from CNS myelination [2,3]. Current evidence notes that OLs originated in jawed vertebrates (gnathosomes) before the divergence between osteichthyses (bony fish) and chondrichthyses (cartilaginous fish) approximately 450 Ma ago. Besides myelination, OLs participate in brain metabolism providing trophic support *via* Glial cell line derived neurotrophic factor (GDNF), Brain-derived neurotrophic factor (BDNF) and also the Insulin like growth factor (IGF-1) [4]. Satellite OLs residing in the gray matter do not participate in myelination but regulate extracellular fluid in the brain.

During primate evolution oligodendrocyte gene expression underwent alterations as a consequence of increasing volume of the primate cortex, as well as changing neurohormonal regulation. The changes impacted the oligodendrocytes by increasing their number, improving their interconnectivity with neurons and other glial cells and altering their physiology in response to neurohormonal regulation. In general, oligodendrocytes could become more supportive for the work of neurones. According to paleoanthropological studies primate hominin lineages underwent genetic, regulatory and structural alterations to the brain [5]. Genetic evolution in hominin and chimpanzee lineages introduced several changes (i.e. chromosomal reorganisation, DNA fragment erasures and replications) after the divergence of hominin and chimpanzee lineages from their last common ancestor 6.5–7.5 Ma [5]. Furthermore, the *KRAB-ZNF* gene that has contributed to primate brain evolution, is especially represented in human and chimpanzee lineages [6]. Brain evolutionary continuity is further exhibited in humans, bonobos and chimpanzees having evolved higher serotonergic input in the infragranular layers of prefrontal cortical areas 9 and 32 [7] which has been speculated may delay gratification response and increase behavioural inhibition [8,9]. Given the evolutionary and genetic similarity between modern humans and the great apes, it is remarkable that the former is vulnerable to numerous neurological and psychiatric pathologies [10]. Unfortunately, there is yet insufficient statistical information regarding the rate and onset of neural disorders in great apes to ascertain whether various neurological and psychiatric pathologies occur only in humans [11]. However, in respect to oligodendrocyte evolution, it is perhaps not unexpected that these glial cells would have also undergone concomitant “genetic alterations” along with modifications in neural cytoarchitecture and cortical folding in ancestral hominins [12, 13].

While deleterious alteration in oligodendrocyte activity has been unequivocally shown in several human brain disorders it is unclear what role evolution has played. Berto *et al*. [12] have proposed that there is greater axonal connectivity of the hominin brain when compared to the non-human primate brain. However, this difference in “human-specific oligodendrocyte genes” [12] does not explain current human susceptibility to neurological and psychiatric pathologies. Recent theories on altered neurohormonal regulation [14,15] provide a more feasible explanation into this evolutionary puzzle. According to paleogeological estimates, the African environment became drier during the Miocene period (ended~5.3 Ma) resulting in a reduction in forests and a concurrent expansion in savannah. This ecological transformation prompted changes in food procurement patterns in early *Homo* such as endurance hunting leading to modifications in human morphology such as loss of body hair, increase in eccrine glands, slow twitch muscles, swivel hips, increase in femur length and bipedal stride and a fully developed foot arch [16,17]. These changes became supported by natural selection due to their enhancing fitness value [18]. Furthermore, endurance hunting resulted in recruiting dopamine for enhancing thermoregulation mechanisms to manage thermal stress [19, 20]. It has been posited that increasing physical activity levels (PAL) and subsequent changes in thermoregulatory mechanisms from *Homo erectus* onwards may have altered calretinin regulation of GABAergic hippocampal interneurons in cortical and subcortical regions which are vulnerable to hyperthermia [21]; second, this evolutionary trade-off may have exposed the human lineage to increasing neurological and psychiatric pathologies, such as schizophrenia. Moreover, increasing temperature in the hippocampus, a site of oligodendrocyte activity, may deleteriously alter GABA mechanisms in pyramidal cells, increasing the risk of “hippocampal excitability” [21,22,23]. For example, the hippocampus in individuals with schizophrenia may reveal volumetric deficits in CA2/3 and CA4/dentate gyrus areas, as well as altered connectivity, suggestive of impaired myelination [24]. Next, oligodendrocytes show a disproportional bias for myelinating inhibitory interneurons (i.e. GABAergic interneurons) in the neocortex [25], thereby increasing the risk for impaired myelination to these interneurons due to possible evolutionary trade-offs, as discussed earlier.

Human brains are approximately three times bigger than chimpanzee, and early human ancestors’ (*Australopithecinae, Homo habilis*), while they have only twice as many cortical neurons as chimpanzees [26]. This means that human neuronal density in the cortex is only about 2/3 of that of a chimpanzee. The rest of the human cortex’s volume is made mostly of glial cells. In the human cortex approximately 75 % of non-neuron cells are OLs. Increased neuronal activity enhances myelination while myelination requires large quantities of protein to be available during a short time during which the complex mechanism of myelin sheet production occurs. Thus, both roles of OLs – regulation of trophic processes and myelination – became enhanced during the evolution of humans that implied increased reliance on neural processing of information and improved acquisition of high-quality foods [27]. This found its expression in the increased proportion of OLs, and other glial cells, to neurons in the human cortex. This quantitative enhancement of the presence of OLs in the human brain highlights the fact that the substrate of human behaviours is not just the “digital” neuronal connectivity but also the overall physiological environment in which the brain operates.

## Structure, ontogeny and the role of oligodendrocytes

**Fig. 1:**
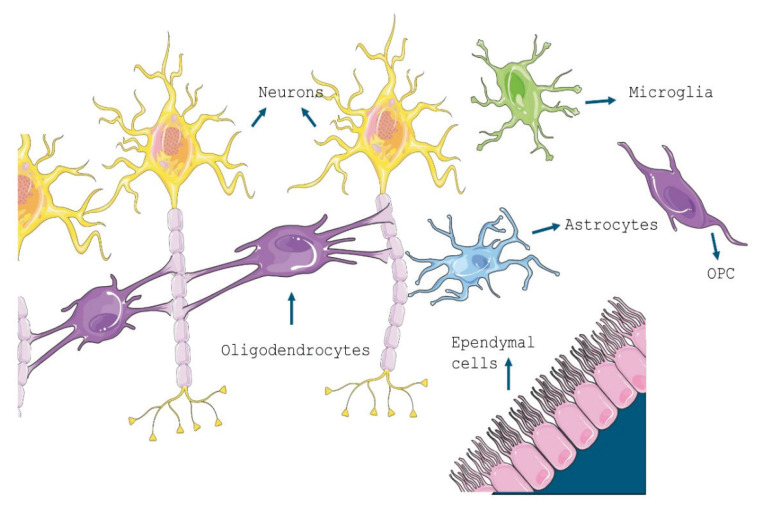
Schematic illustration of glial cells (microglia, astrocytes, ependymal cells and the oligodendrocytes) of the central nervous system in a mammalian brain. Astrocytes-provide structural support, help in the formation of the blood-brain barrier; microglia-scavenge pathogens, dead and dying cells; ependymal cells-produce cerebrospinal fluid that cushions neurons; oligodendrocytes-insulation, myelination, protection and support; oligodendrocyte progenitor cells (OPC) also called NG2glia-remyelination, synaptic plasticity, immunomodulation. Image is created/procured from Servier Medical Art by Servier under a Creative Commons Attribution 3.0 Unported license.

**Fig. 2:**
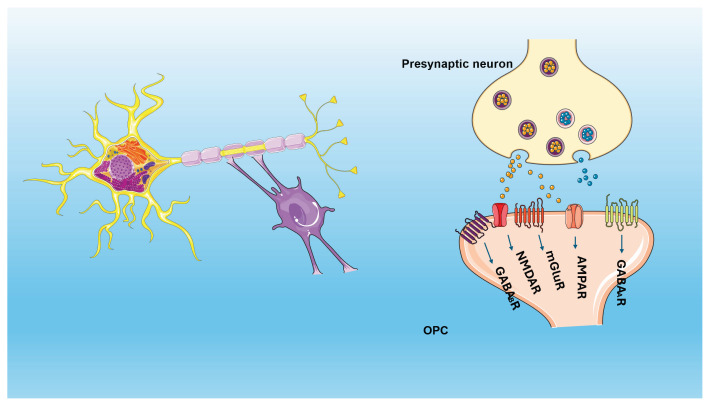
Schematic illustration of oligodendrocyte/OPCs with neuronal cells. Glutamatergic/GABAergic neuron-to-OPC synaptic interactions exist in different brain regions, such as the hippocampus. Postsynaptic responses in OPCs are mediated by glutaminergic receptors (mGluR, AMPA & NMDA) *via* the activation of presynaptic glutaminergic neurons, and GABAergic receptors get activated through presynaptic GABAergic neurons leading to enhanced post-synaptic response. Image is created/procured from Servier Medical Art by Servier under a Creative Commons Attribution 3.0 Unported license.

Glial cells comprise of astrocytes, ependymal cells, microglia and oligodendrocytes in the Central Nervous System (CNS) (Fig. 1). Oligodendrocytes are responsible for producing and maintaining myelin, a lipid-enriched highly organised multi-layer membrane structure that surrounds and insulates axons [28,29,30]. This insulation helps speed up action potentials along the axon and allows for saltatory conduction [30,31]. The structure of OLs consists of a cell body, several processes, and several branches. The cell body contains the nucleus and other organelles, while the processes extend from the cell body and wrap around the axons to form myelin sheaths [32]. The branches of OLs can also communicate with other cells in the nervous system, including neurons (Fig. 2), astrocytes, and microglia [33,34].

Oligodendrocytes develop from a specific precursor cell called the oligodendrocyte progenitor cell (OPC), which is present in the developing (CNS). oligodendrocyte progenitor cells are produced from the ventral ventricular zone of the neural tube during embryonic development [35]. These precursor cells migrate throughout the developing CNS and differentiate into mature OLs capable of producing myelin [36].

Research has identified that OPCs can also be produced from another type of cells, such as neural stem cells and glial-restricted progenitor cells, which can differentiate into both neurons and glial cells. Furthermore, it has been shown that OPCs can be produced from astrocyte subpopulation, known as NG2 glia, which can differentiate into OPCs in response to some specific signals [37,38]. Overall, the origin of OLs is a complex process that involves multiple pathways and cell types and is still being studied and understood by researchers.

The uniform density and spacing of these cells are defined by these dynamic and exploratory activities, which are self-repulsive toward other OPCs. After OPCs have gone within their destined regions, their next courses can be extremely diverse. Some OPCs remain in the precursor state, while others differentiate into myelin-forming oligodendrocytes [39]. Apoptosis eliminates the surplus cells produced in order to guarantee that the number of OLsand axons to be myelinated is equal [40]. Maturating OPCs undergo many morphological stages of development and express a number of cell-specific marker proteins and lipids [41]. oligodendrocyte progenitor cells can be easily recognised by the expression of specific markers such as platelet-derived growth factor alpha receptor (PDGFR2α), polysialylated form of neural adhesion molecule (NCAM), and Olig2 [41,42]. During the developmental process, these cells undergo a sequence of molecular and morphological changes *via* several signalling pathways and transcription factors, including Sonic Hedgehog, Notch, and Olg1/2 [43,44,45]. The migration of OPCs is mediated by various signalling molecules and adhesion molecules, including laminin and integrin receptor α6β1. Once OPCs reach the destined area they differentiate into mature OLs capable of myelinating axons. The main functions include myelination [30], axonal support [46], ion homeostasis [47], and neuroprotection [48].

Oligodendrocytes and their progenitors are directly associated in membrane and metabolic interactions with neurons during the distinct stages of degradation and regeneration of the myelin sheath, driven by the dynamic and changing expression of several transcription factors [49]. Proper myelination is required for the normal formation and evolution of neural connections, as well as brain functional response to environmental changes. Myelination continuously reshapes neuron/oligodendrocyte interactions based on a variety of parameters, including learning, social relationships, and emotional cues [50]. These stressors can cause epigenetic changes that affect the physiology and functionality of precursors and Ols.

The topic of myelinating OL support of axons has been extensively reviewed elsewhere [51,52], so here we focus on more recent evidence of how the myelin-producing OLs are directly damaged in AD and the associated consequences. (Fig. 2)

## AD and Oligodendrocyte dysfunction

AD is a degenerative disorder that affects mainly the elderly neuro population [53]. AD exhibits multiple neurobehavioral deficits [54,55]. However, this is majorly characterised by the proteinopathies [56,57]. Pathologically, AD reveals the existence of extracellular amyloid β (Aβ) and intracellular neurofibrillary tangles (NFTs), with significant degeneration of white matter and myelin loss [58,59]. In general, the extracellular abnormalities of amyloid cascade predominate [60]. The multifaceted nature of AD poses difficulties in treatment. Consequently, understanding the underlying aetiology and progression of AD may enable scientists to develop improved therapies.

Initially, AD is considered to primarily affect grey matter which progresses to cause abnormalities in white matter and demyelination is well documented [61]. Myelin is an essential component required for homeostasis; myelin defects can drive amyloid deposition, and lead to the increase of the amyloid-generating machinery within axonal swellings and elevate the cleavage of cortical amyloid precursor protein (APP) [60]. Moreover, latest scientific data have identified the pro-inflammatory activity of Aβ on microglia [57,62] astrocytes [63,64] and a little is known regarding OLs. However, OLs express certain types of cell surface receptors such as TLRs [65], RAGE [66] which can interact with Aβ [67]. Initially it was theorized that AD might be a part of the response to age-related myelin breakdown. Interestingly myelin defects or injuries have been shown to be the drivers of amyloid deposition in AD [68], so OLs remain an area of medical interest in AD, given their central role in myelin production and axonal support. Higher neuronal activity may cause OLs to alter myelin development and distribution, influencing action potentials across the neurons [69]. However, in AD pathogenesis where there is a progressive neuronal and axonal dysfunction is directly correlated with the amount of myelin produced by OLs. Ongoing deficits in myelin and OL activity ultimately affect cognition and memory. Oligodendrocyte vulnerability revealed myelin breakdown in various forms of AD. This myelin loss appears to be the first step in early AD even before the appearance of amyloid plaques [70]. Alzheimer’s disease commences in the temporal-entorhinal cortex in cognitively normal individuals. The majority of late-onset/sporadic AD commence due to the accumulation of amyloid proteins in the lateral entorhinal cortex, *via* limbic-neocortical trajectory, where the data from different regions of neocortex converge on the entorhinal gateway to the hippocampus [71].

Various clinical and preclinical studies investigated OL changes in AD. A study on a mice model revealed an increase in amyloid plaque load in hippocampal white matter (alveus) and cortex at 6 months of age. Moreover, control-mice and myelin mutant mice did not show plaque pathology in the alveus at this age [72]. Another study analysed cells of the oligodendrocyte lineage in a mouse model with chronic plaque deposition (APP PS1 mice) and also, in samples from human patients. It was found that APPPS1 mice had a larger number of cells of the OL lineage (Olig2+ cells), but a postmortem human AD cortex had fewer Olig2+ cells. Their findings show that OPCs negatively respond to amyloid plaque deposition in an AD-mice model and in human AD, albeit, with different results. Surprisingly, putative repair mechanisms from freshly formed OL are evident in APPPS1 animals, although, a comparable response of OPCs appears to be severely limited in the later phases of human AD disease [73].

Apart from the mutations in genes such as presenilin-1 (PS1), presenilin-2 (PS2), APOE [74], oxidative stress [75,76,77] in various forms is known to contribute to AD pathogenesis, acting as a demyelinating factor that leads to neuronal damage, glial cell damage and neurodegeneration [78,79]. OL’s develop from OPCs, which can remove Aβ by phagocytosis and autophagy [80]. Alzheimer’s disease reduces the number of OLs and OPCs, making it difficult to clear Aβ in the brain. As previously specified, OLs are also engaged in the neurodegenerative process, and their number falls with the course of Alzheimer’s disease.

Moreover, one widely accepted theory regarding OL damage in AD is the influence of oxidative stress, which is generated by numerous factors. To compensate for this, the adult CNS produces OPCs which differentiate into myelinating OLs. Even though, OPCs make up 5 % of the parenchymal cells, they serve as a backup for OL loss or demyelination, and they also play an imperative role in cognitive processes like learning and memory. Evidence suggests that OPC dysfunction, including lack of differentiation, contributes to the advancement of AD [81]. Neurodegeneration has been linked to oxidative stress, which might impact OPC plasticity due to its high metabolic needs and weak antioxidant activity [82].

OL are the principal iron containing cells of the brain. However, they have low levels of glutathione, glutathione peroxidase, and mitochondrial superoxide dismutase. The ROS-activated matrix metalloproteases induce OL dysfunction by destroying the extracellular matrix, eventually damaging the differentiation. OLs of PS1 mutant knock-in mice were found to be more susceptible to glutamate, APP toxicity [83,84], and lead to OL death by promoting calcium dysregulation. This study also revealed that a disease-causing PS1 mutation had a negative effect on OLs that caused white matter destruction in AD, there by contributing to cognitive impairment [83].

Oxidative stress also damages OPC differentiation by reducing the gene levels such as SHH, SOX10, and HDAC3 that promote OL differentiation. This has been previously confirmed by cell-culture experiments where the pre-oligodendrocytes showed a high degree of sensitivity to oxidative stress and low glutathione content [82]. Moreover, mutations of PS1 predisposed mouse oligodendrocyte precursor cells to amyloid-induced alterations *in vitro* [84].

Furthermore, microglia activation and release of inflammatory cytokines may contribute to myelin damage in AD. Microglia also play a crucial role in removing toxins from the brain, which is vital for remyelination. When Aβ accumulates in the brain, microglia get activated, which reduces the quantity of neurotoxic soluble Aβ in the brain.

Although, damage to white matter is a symptom found in AD patients, it is unclear whether and how OLs are damaged in AD, or whether white matter abnormalities contribute to cognitive failure. However, changes in OLs have been demonstrated and observed in various transgenic animal models such as APP/PS1/5XFAD,3xTg [83, 85, 86, 87]. In all these models the OL number decreased drastically along with the destruction of myelin. It is important to note that the ApoE4 allele is associated with a higher level of myelin destruction in AD. ApoE, a known risk factor for the disease, is involved in the transport and recycling of endogenously produced brain lipids, which is essential for myelin formation, maintenance, and repair [88,89]. It has been shown that apoE4 allele carriers have lower amounts of ApoE molecules in serum and brain tissue than non-carriers [89]. The Apo E4 genotype reduces myelin production in the human brain and increases age-related myelin degradation.

Humans exhibit a disproportional increase of prefrontal white matter in comparison to other primates, which may have an effect on myelination in AD. A study on genetic mice with mainly unmyelinated cortical axons found that the plaque burden in the alveus of the hippocampus was significantly higher. This study demonstrated the inhibitory effect of healthy myelin and appropriate myelin ensheathment on plaque development [72], as well as myelin deficits which alter microglia responses. Another study on human post-mortem AD brains identified a significant reduction in the mean nuclear diameter of neurons and OLs in the temporal lobe [90].

Numerous studies have reported the toxicity of Aβ to OLs [85], Erten-Lyons *et al*. [91]. The composition of myelin and its architecture may seem to be an early target for toxic amyloid and other misfolded proteins [60]. Additionally, amyloid-β damages mitochondrial DNA leading to the subsequent NF-κB and AP-1 activation, even though these Aβ molecules rarely exist in the white matter of AD brains. Only the levels of soluble Aβ are increased in white matter [92, 93]. However, eradication of amyloid plaques in various clinical trials failed to prevent the cognitive decline associated with neurodegeneration. This indicates that amyloid beta molecules need to be targeted in the early stages of AD. Similarly, tau fibrils also cause cognitive decline in AD. Physiological tau phosphorylation is required for the stabilisation of microtubules. However, the hyperphosphorylation would result in the formation of neurofibrillary tangles in neurons, astrocytes, microglia, and OLs [94]. This phosphorylated tau in grey matter is related to white matter abnormalities and subsequent demyelination [95]. Therefore, phosphorylated tau can be a predictor of white matter deficits. The amount of myelin in the brain and the integrity of the myelin sheath deteriorates with age.

Myelin content peaks in middle age and then gradually diminishes in later years [96]. There is evidence that age-related cognitive impairment is linked to white matter abnormalities [97], which could be caused by significant demyelination and OL loss. Age-linked alterations in the integrity of white matter are connected with cognitive deterioration in healthy elderly people [98]. Gene expression in OLs has been known to undergo further dramatic acceleration in the human lineage compared to neurons and primitive gene expression studies were likely underpowered to detect these non-neuronal expression alterations [12].

## Markers of oligodendrocytes/myelin

OL originate as migratory and mitotic precursors, progress to progenitors, and eventually mature into postmitotic myelin-producing cells. The expression of specific markers, recognised by a list of cell specific antibodies. Majority of these markers were first found in tissue cultures, minority of them are specific to the components of myelin. The myelination process is accompanied by alteration in the cell surface antigens expression also called antibodies. Some of the progenitor markers include nestin, proteolipid protein, platelet derived growth factor alfa receptor, ganglioside GD3, transferrin, S100 proteins and glutamine synthetase. While the major myelinating mature OL specific markers are given below:

## Glycolipids

Sphingolipids galactosylceramide (GalCer), sulfatide (ST) and sphingomyelin (SM) are essential for myelin stability and function. Oligodendrocytes express specific glycolipids such as (GalCer) and ST remain present on the surface of mature oligodendrocytes. GalCer and ST are synthesized mostly from C22–C24 ceramides, generated by Ceramide Synthase 2 (CerS2). Loss of CerS2 in myelinating cells resulted in greatly reduced C22–C24 sphingolipids and increased C16–C18 sphingolipids in myelin. This was associated with an overall reduction in myelin sheath thickness, and a decrease in the proportion of myelinated axons [99].

## RIP Antigen

The RIP monoclonal antibody is commonly used for immunohistological detection of mature OLs. The RIP antigen was recognised as 2′,3′-cyclic nucleotide 3′-phosphodiesterase (CNPase), a non-compact myelin protein. The antibody was directed toward antigens found in mature oligodendrocyte cytoplasm in order to create a reliable marker of somata and myelinating processes, rather than myelin sheath components. This marker can serve to determine biochemical subtypes of oligodendrocytes [100].

## Carbonic anhydrase II (CAII)

Vast majority of CAII is localised, in oligodendrocytes and myelin sheaths. An increase in the CA II activity was reported in the developing rat brain during the period of myelination and a decrease of the enzyme activity was found in autopsy material of human leukodystrophies. CAII covers all stages of the lineage and is also a marker of adult oligodendrocytes [101].

## NI-35/250 proteins

These are trans membranous proteins mostly found in OL and myelin of mammals. These are potent inhibitors of axonal regrowth in pathological conditions. [41].

## Specific myelin proteins

Genes encoding the specific myelin proteins are expressed at different stages of the OL differentiation and maturation. 29,39-Cyclic nucleotide-39-phosphohydrolase (CNP), myelin basic protein (MBP), PLP/DM-20, myelin-associated glycoprotein (MAG), and myelin/oligodendrocyte glycoprotein (MOG) genes as well as other minor myelin proteins [41].

## Conclusion

Myelin damage probably precedes the onset of pathological alterations such as amyloid plaques and neurofibrillary tangles. Evidence suggests that amyloid fibrils destroy the myelin hindering saltatory transmission thereby leading to cognitive decline. Despite AD being a heterogenous disease with multiple causes and multiple targets, AD-like pathology is seen at low levels in the entorhinal cortex of ageing primates (monkeys, chimpanzees) but, it emerges faster in humans in the entorhinal cortical region due to the immense amount of data overloading the memory system during the process of ageing. Furthermore, this region of the brain contains clusters of myelinated neurons, which are in turn loaded with mitochondria and are highly metabolic active, so they continue to generate free radicals as part of generating ATP. This increases susceptibility to oxidative stress and leads to the seeding of amyloid plaques, neuronal damage, myelin, and OL damage.

## Abbreviations

AD: Alzheimer’s disease
AP-1: Activator protein-1
APOE: Apolipoprotein E
APP: Amyloid precursor protein
ATP: Adenosine triphosphate
Aβ: Amyloid beta
BDNF: Brain-derived neurotrophic factor
CA: Cornu Ammonis
CNS: Central Nervous System
GABA: Gamma-aminobutyric acid
GDNF: Glial cell line derived neurotrophic factor
IGF-1: Insulin like growth factor
NCAM: Neural adhesion molecule
NFTs: Neurofibrillary tangles
NF-κB: Nuclear factor κ-light-chain-enhancer of activated B cells
Ols: Oligodendrocytes
OPC: Oligodendrocyte progenitor cell
PAL: Physical activity levels
PDGFR2α: Platelet-derived growth factor alpha receptor
PS: Presenilin
RAGE: Receptor for Advanced Glycation End products
TLRs: Toll-like receptors
